# Habitat Temperature and Precipitation of *Arabidopsis thaliana* Ecotypes Determine the Response of Foliar Vasculature, Photosynthesis, and Transpiration to Growth Temperature

**DOI:** 10.3389/fpls.2016.01026

**Published:** 2016-07-25

**Authors:** William W. Adams, Jared J. Stewart, Christopher M. Cohu, Onno Muller, Barbara Demmig-Adams

**Affiliations:** Department of Ecology and Evolutionary Biology, University of Colorado BoulderBoulder, CO, USA

**Keywords:** *Arabidopsis thaliana*, ecotypic variation, foliar vasculature, phloem, photosynthesis, temperature acclimation, transpiration, xylem

## Abstract

Acclimatory adjustments of foliar vascular architecture, photosynthetic capacity, and transpiration rate in *Arabidopsis thaliana* ecotypes (Italian, Polish [Col-0], Swedish) were characterized in the context of habitat of origin. Temperatures of the habitat of origin decreased linearly with increasing habitat latitude, but habitat precipitation was greatest in Italy, lowest in Poland, and intermediate in Sweden. Plants of the three ecotypes raised under three different growth temperature regimes (low, moderate, and high) exhibited highest photosynthetic capacities, greatest leaf thickness, highest chlorophyll *a*/*b* ratio and levels of β-carotene, and greatest levels of wall ingrowths in phloem transfer cells, and, in the Col-0 and Swedish ecotypes, of phloem per minor vein in plants grown at the low temperature. In contrast, vein density and minor vein tracheary to sieve element ratio increased with increasing growth temperature – most strongly in Col-0 and least strongly in the Italian ecotype – and transpirational water loss correlated with vein density and number of tracheary elements per minor vein. Plotting of these vascular features as functions of climatic conditions in the habitat of origin suggested that temperatures during the evolutionary history of the ecotypes determined acclimatory responses of the foliar phloem and photosynthesis to temperature in this winter annual that upregulates photosynthesis in response to lower temperature, whereas the precipitation experienced during the evolutionary history of the ecotypes determined adjustment of foliar vein density, xylem, and transpiration to temperature. In particular, whereas photosynthetic capacity, leaf thickness, and foliar minor vein phloem features increased linearly with increasing latitude and decreasing temperature of the habitats of origin in response to experimental growth at low temperature, transpiration rate, foliar vein density, and minor vein tracheary element numbers and cross-sectional areas increased linearly with decreasing precipitation level in the habitats of origin in response to experimental growth at high temperature. This represents a situation where temperature acclimation of the apparent capacity for water flux through the xylem and transpiration rate in a winter annual responded differently from that of photosynthetic capacity, in contrast to previous reports of strong relationships between hydraulic conductance and photosynthesis in other studies.

## Introduction

Linking natural variation in the genome and in gene expression to phenotypic variability in response to different environmental factors is a goal among researchers working on the model species *Arabidopsis thaliana* (Alonso-Blanco and Koornneef, 2000; Koornneef et al., 2004; Tonsor et al., 2005; Alonso-Blanco et al., 2009; Koornneef and Meinke, 2010; El-Soda et al., 2014; Provart et al., 2016). Clinal variation in the genome, growth parameters, multiple life history traits, and responses to, and tolerance of, low (freezing and chilling) and high temperature, drought, and irradiance (ultraviolet, visible, and far-red) in this species has been documented in numerous studies and typically presented in the context of a geographic gradient in one or more environmental factors (e.g., temperature, photoperiod, precipitation). Altitude has been the primary geographic variable in some studies (Ward and Strain, 1997; Montesinos-Navarro et al., 2011, 2012; Picó, 2012; Wolfe and Tonsor, 2014; Botto, 2015; Luo et al., 2015; Singh et al., 2015; Vidigal et al., 2016), whereas a combination of altitude and distance from the ocean was examined in a few others (Montesinos et al., 2009; Lewandowska-Sabat et al., 2012; Kang et al., 2013). Variation (especially genetic) along longitudinal gradients has also been considered in a number of studies (Schmuths et al., 2004; Beck et al., 2008; Reininga et al., 2009; Panthee et al., 2011; Samis et al., 2012; Zuther et al., 2012; Brachi et al., 2015), and edaphic, biotic, and/or environmental factors were shown to correlate with genomic variation and life history traits in several studies (Lasky et al., 2012; Brachi et al., 2013; Manzano-Piedras et al., 2014). The vast majority of studies focused on variation in traits that vary with latitude of origin (Li et al., 1998; Stenøien et al., 2002; Caicedo et al., 2004; Stinchcombe et al., 2004, 2005; Alonso-Blanco et al., 2005; Hannah et al., 2006; Li et al., 2006; Hopkins et al., 2008; Kipp, 2008; Tonsor et al., 2008; Zhen and Ungerer, 2008; Jansen et al., 2010; Chiang et al., 2011; Nägele et al., 2011, 2012; Zuther et al., 2012; Barah et al., 2013; Debieu et al., 2013; Distelbarth et al., 2013; Kurbidaeva et al., 2015), most likely because both photoperiod and temperature vary extensively along north-south transects.

**FIGURE 1:**
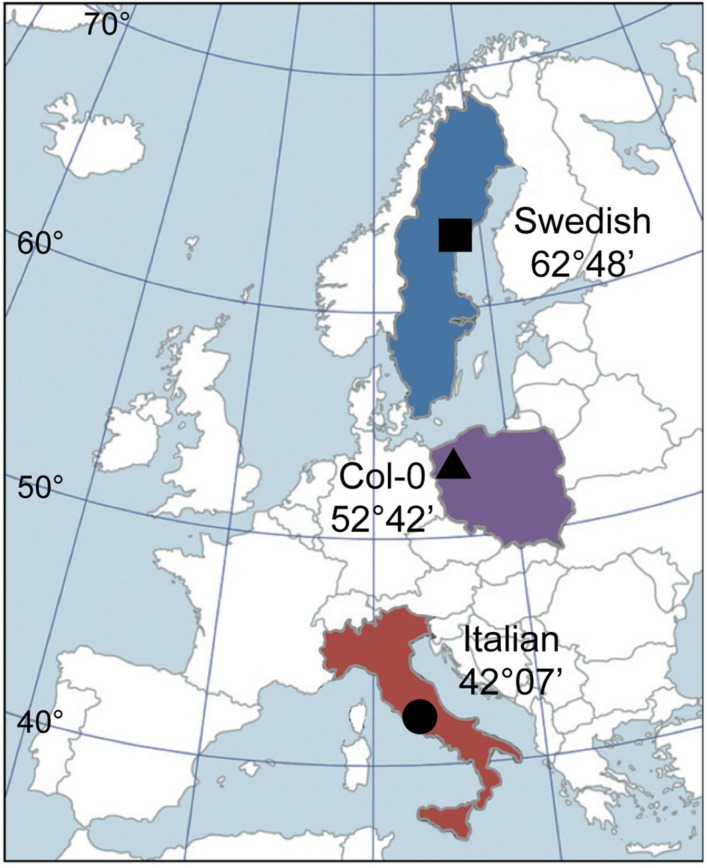
**Map showing the locations and latitudes from which the three ecotypes of *Arabidopsis thaliana* employed in this study originated.** Squares (Swedish ecotype), triangles (Col-0), and circles (Italian ecotype) are used to designate each ecotype in all of the following figures, and the colors are used to illustrate each habitat/ecotype in **Figures 2**, **3**, **5**, and **10**. Redrawn after Ågren and Schemske (2012).

Two European populations of *A. thaliana*, separated by 2298 km of latitudinal distance, have been the focus of collaboration among multiple laboratories. First described by Ågren and Schemske (2012), the southern ecotype stems from Castelnuovo di Porto (42°07′ north latitude) in central Italy and the northern ecotype from Rödåsen (62°48′ north latitude) in central Sweden (**Figure 1**). In an initial 5-year characterization in the field (Ågren and Schemske, 2012), reciprocal transplants established strong local adaptation through differences in fitness (survival, fecundity) between the two ecotypes in each site. Subsequent studies using recombinant inbred lines of the two parental lines examined some of the underlying genetics responsible for those fitness tradeoffs and other life history traits (Ågren et al., 2013; Dittmar et al., 2014; Oakley et al., 2014; Postma and Ågren, 2015). Quantitative trait locus mapping was used to identify genes underlying flowering in the two populations under both vernalizing and non-vernalizing conditions (Grillo et al., 2013) and additional insights into adaptive and maladaptive dominant and recessive genes were gained through crosses and backcrosses using the two parental lines (Oakley et al., 2015). Furthermore, the impact of leaf damage (Akiyama and Ågren, 2012) and germination timing (Akiyama and Ågren, 2014) on fitness was evaluated in the Swedish ecotype in the field. Moreover, the Swedish ecotype is relatively tolerant of freezing stress, whereas the Italian ecotype, with a non-functional C-repeat binding factor 2 (part of the CBF family of transcription factors that play a prominent role in acclimation to low temperature), is much less tolerant of freezing (Gehan et al., 2015). This latter finding led to a wider exploration of the role of CBF modification in the acclimation of multiple *A. thaliana* ecotypes to warmer climates (Monroe et al., 2016).

**FIGURE 2:**
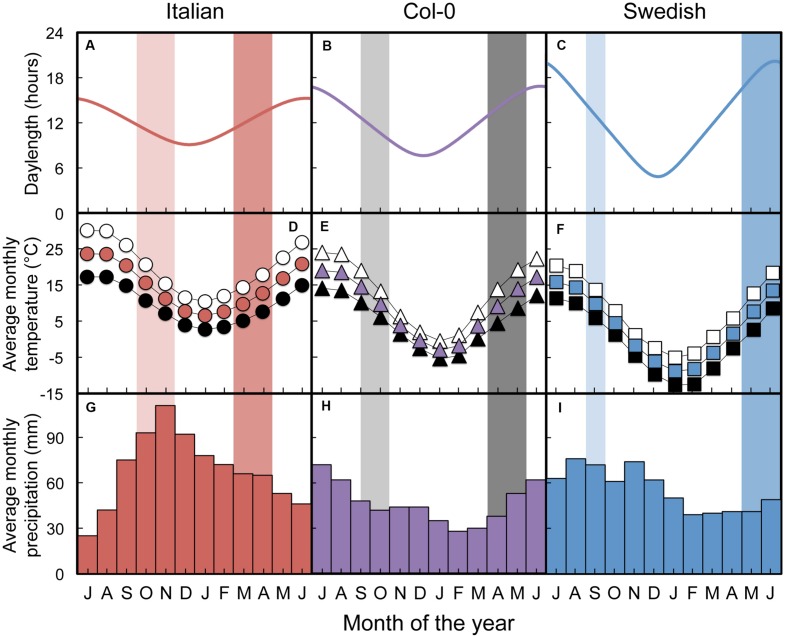
**(A–C) Photoperiod, **(D–F)** average monthly high temperature (open symbols), average monthly temperature (colored symbols), and average monthly low temperature (black symbols), and **(G–I)** average monthly precipitation for the sites from which the Italian **(A,D,G)**, Col-0 **(B,E,H)**, and Swedish **(C,F,I)***A. thaliana* ecotypes were obtained.** The periods of germination (light shade) and bolting through flowering (moderate shade) are also shown behind the data across all three panels for each habitat based upon observations reported by Grillo et al. (2013) for the Italian and Swedish ecotypes. Each year starts with the month of July and ends with the month of June.

**FIGURE 3:**
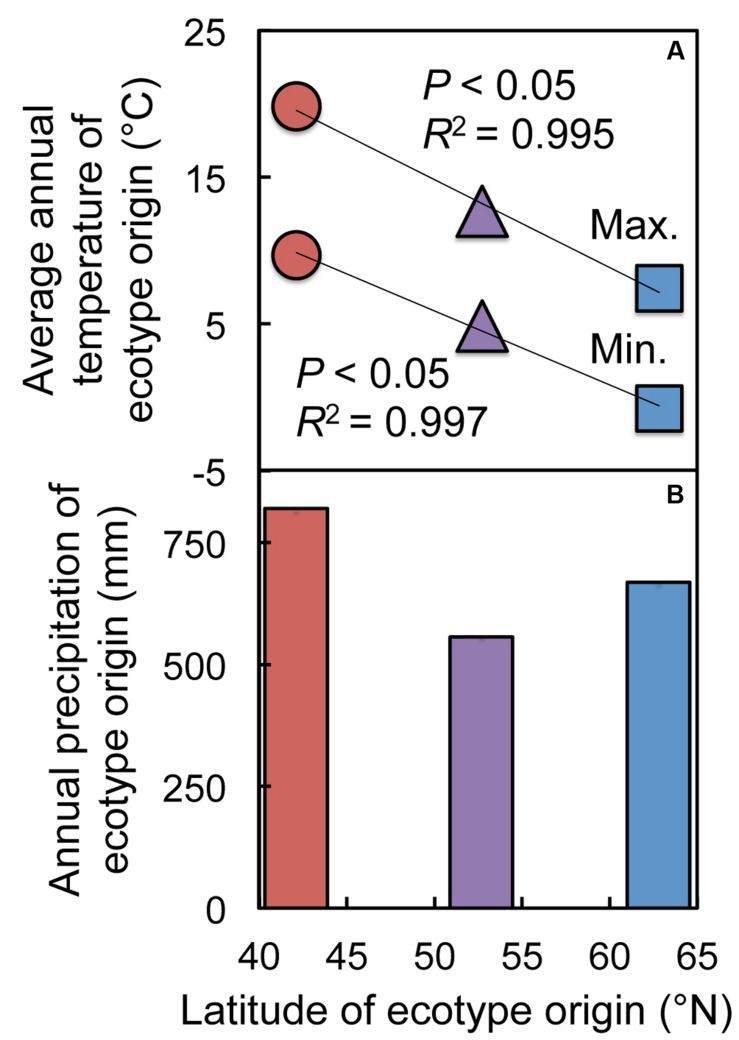
**Average annual **(A)** maximum and minimum temperatures and **(B)** precipitation for the sites from which the Italian (red circles and column), Col-0 (purple triangles and column), and Swedish (blue squares and column) *A. thaliana* ecotypes were obtained**.

From a functional standpoint, both ecotypes were shown to exhibit acclimation to experimental growth temperature and light environment, with more pronounced acclimatory adjustments in chloroplast pigments and tocopherols, photosynthesis, plant and leaf structure, and foliar vasculature (particularly the phloem) in the Swedish compared to the Italian ecotype (Cohu et al., 2013a,b, 2014a; Adams et al., 2014a; Stewart et al., 2015, 2016). The current study was undertaken to place acclimatory adjustments in leaf architecture (and especially foliar vascular features), photosynthetic capacity, and transpiration in response to growth under controlled experimental conditions in growth chambers at three different temperatures (low, moderate, and high) in the context of ecotypic latitude of origin and the environmental conditions prevailing at each respective site of origin. Furthermore, these ecotypes were used to illuminate relationships among various vascular metrics. In addition to the northern and southern European ecotypes from Sweden and Italy, the widely used Columbia (Col-0) line was included as an ecotype from a latitude intermediate to those of the Swedish and Italian ecotypes. Derived from seed collected in the Landsberg an der Warthe region (outside the current city of Gorzów Wielkopolski in Poland; Rédei, 1992; Koornneef and Meinke, 2010; latitude of 52°42′ N from El-Lithy et al., 2004), the Col-0 ecotype stems from a site almost equidistant in latitude between the sites from which the Swedish and Italian ecotypes originate (**Figure 1**); there are 1176 km of latitude between the Italian and Polish sites and 1122 km of latitude between the Polish and Swedish sites. All three habitats of origin are low altitude sites near major bodies of water: the Swedish site is just over 80 m above sea level and less than a kilometer from the Gulf of Bothnia (Baltic Sea), the Polish site is just short of 60 m above sea level and approximately 160 km from the Baltic Sea, and the Italian site is approximately 225 m above sea level and 35 km from the Tyrrhenian Sea (Mediterranean Sea). The symbols pinpointing the location of the sites of origin (a circle for the Italian site, a triangle for the Polish site, and a square for the Swedish site; **Figure 1**) are used to designate the ecotypes originating from these three sites in all subsequent figures. In addition, the colors used to identify the countries of origin (red for Italy, purple for Poland, and blue for Sweden) are also used to identify the three sites/populations in **Figures 2**, **3**, **5**, and **10**.

These three ecotypes of *A. thaliana* thus allow for characterization of phenotypic plasticity that has presumably evolved in response to conditions experienced by these distinct populations from the three sites – with otherwise remarkably similar features – along an extensive latitudinal gradient. In order to evaluate the acclimation potential within each ecotype, plants were grown in growth chambers at different temperatures spanning more than 20°C. Given the many generations over which the Col-0 line has been maintained under controlled laboratory conditions since its original collection in 1955 (Rédei, 1992), there was a possibility that this ecotype would no longer exhibit traits aligned with its habitat of origin. However, based on the results of the present study, this does not seem to be the case for the evaluated features.

## Materials and Methods

### Plant Material, Climatological Information, and Growth Conditions

Three ecotypes of *Arabidopsis thaliana* (L.) Heynhold were investigated. Seed of Col-0 was obtained from *The Arabidopsis Information Resource*^1^ for comparison with the Swedish and Italian ecotypes (see Stewart et al., 2015 for a detailed description of the latter two ecotypes). Climatological information on the habitats from which the three ecotypes originated was obtained from http://www.worldclim.org/ (Hijmans et al., 2005) and the photoperiods prevailing in each site were estimated from the US Naval Observatory’s website^2^, all of which are summarized in **Figures 2** and **3**. Plants were grown in 2.8 l pots (one plant per pot) containing Canadian Growing Mix 2 (Conrad Fafard Inc., Agawam, MA, USA) under controlled conditions in growth chambers (Conviron E-15, Winnipeg, Canada), receiving water daily and nutrients every other day. Multiple plants of each ecotype were grown under three independent day/night air temperature regimes of 8°C/12.5°C (low or cool), 25°C/20°C (moderate), and 35°C/25°C (high or hot) under a common photon flux density of 400 μmol m^-2^ s^-1^ (provided by a mixture of cool white fluorescent bulbs and incandescent bulbs) during a 9 h photoperiod as described in Cohu et al. (2013b) and Stewart et al. (2016). Through evaluation of the response of some of these metrics to other combinations of temperature and light, it was determined that the greatest impact of temperature on the metrics examined could be seen at this intermediate light intensity (a higher growth light intensity resulted in saturation of many of the responses so that the differential impact of temperature was less distinguishable; see Cohu et al., 2013b). The experimental design thus consisted of three ecotypes by three growth temperature regimes, although the majority of data presented focus on the low and high temperature regimes (three ecotypes by two growth temperature regimes). In addition to evaluating the various metrics in relationship to each other, some metrics were also evaluated in relationship to the latitude of ecotypic origin as well as the temperatures and precipitation experienced by each ecotype in its natural habitat. The radiative heating experienced by the plants when lights were on in the growth chambers resulted in leaf temperatures (determined with a fine thermocouple; Wescor TH-65 meter, Logan, UT, USA) of 14°C, 28°C, and 36°C during the photoperiods. These daytime leaf temperatures are used to identify the three temperature regimes throughout the manuscript and are also used as the independent variable in **Figure 7**. Fully expanded leaves from three to five non-flowering plants were evaluated for each ecotype and growth temperature.

### Leaf Metrics

**FIGURE 4:**
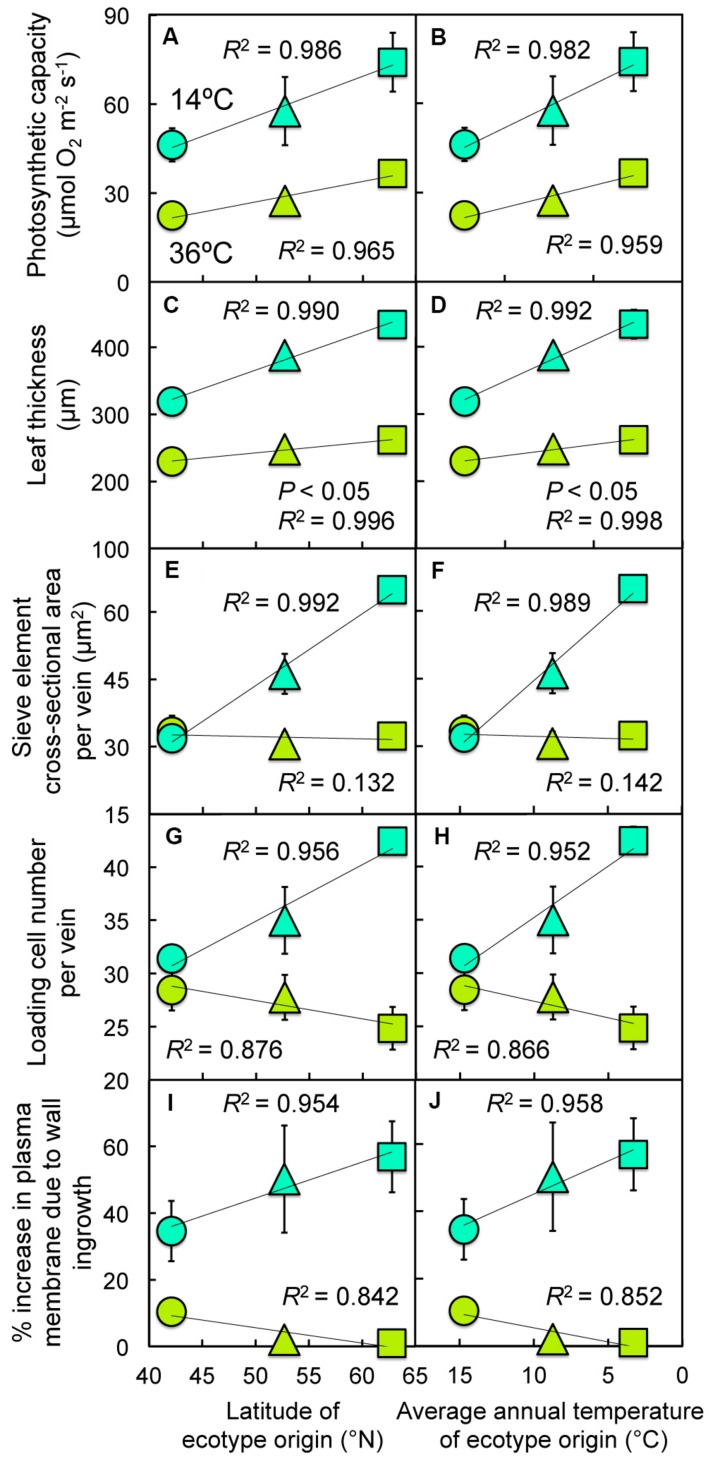
**Relationship between **(A,B)** photosynthetic capacity (light- and CO_2_-saturated rate of photosynthetic oxygen evolution), **(C,D)** leaf thickness, **(E,F)** cross-sectional sieve element area per minor vein, **(G,H)** number of cells (phloem parenchyma, companion, and sieve element) contributing to the loading of sucrose per minor vein, or **(I,J)** the estimated percent increase in cell membrane length due to wall ingrowth in minor vein phloem transfer cells and **(A,C,E,G,I)** latitude of ecotype origin or **(B,D,F,H,J)** average annual temperature at the site from which each ecotype was obtained for leaves of Italian (circles), Col-0 (triangles), and Swedish (squares) *A. thaliana* ecotypes grown at cool (14°C = blue-green symbols) or hot (36°C = olive-green symbols) leaf temperature.** Colors inspired by the relative foliar level of chlorophyll *a* to chlorophyll *b* under each growth temperature regime (see **Figure 7H**).

**FIGURE 5:**
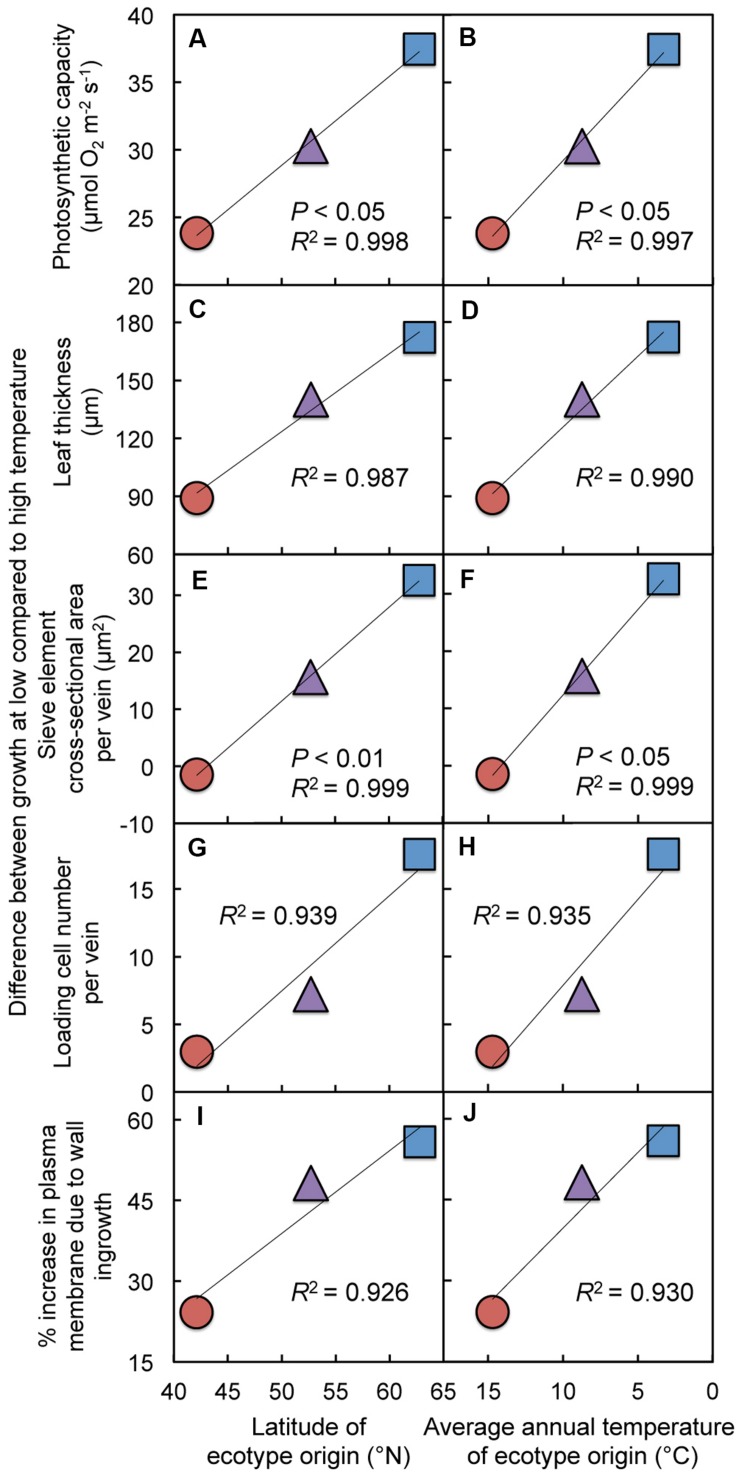
**Differences between growth at high (36°C) compared to low (14°C) temperature in **(A,B)** photosynthetic capacity, **(C,D)** leaf thickness, **(E,F)** sieve element cross-sectional area per minor vein, **(G,H)** number of cells (phloem parenchyma, companion, and sieve element) contributing to the loading of sucrose per minor vein, and **(I,J)** the estimated percent increase in cell membrane length due to wall ingrowths in minor vein phloem transfer cells for leaves of Italian (red circles), Col-0 (purple triangles), and Swedish (blue squares) *A. thaliana* ecotypes as a function of **(A,C,E,G,I)** the latitude of ecotype origin or **(B,D,F,H,J)** average annual temperature at the site from which each ecotype was obtained**.

**FIGURE 6:**
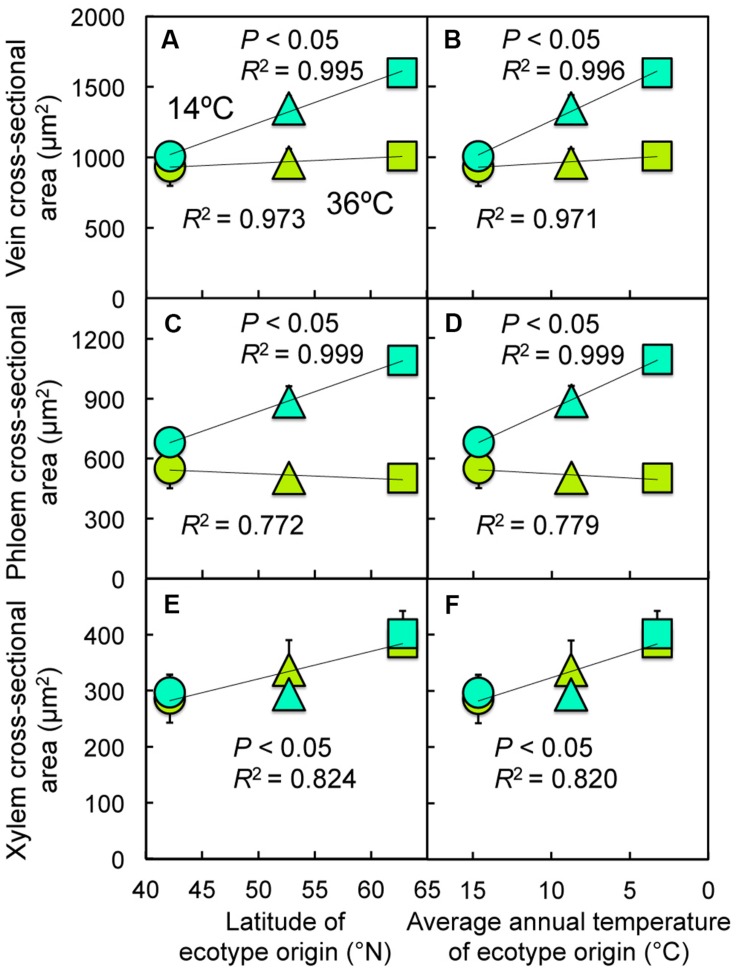
**Relationship between **(A,B)** cross-sectional area per minor vein, **(C,D)** cross-sectional area of phloem per minor vein, or **(E,F)** cross-sectional area of xylem per minor vein and **(A,C,E)** latitude of ecotype origin or **(B,D,F)** average annual temperature at the site from which each ecotype was obtained for leaves of Italian (circles), Col-0 (triangles), and Swedish (squares) *A. thaliana* ecotypes grown at cool (14°C = blue-green symbols) or hot (36°C = olive-green symbols) leaf temperature**.

**FIGURE 7:**
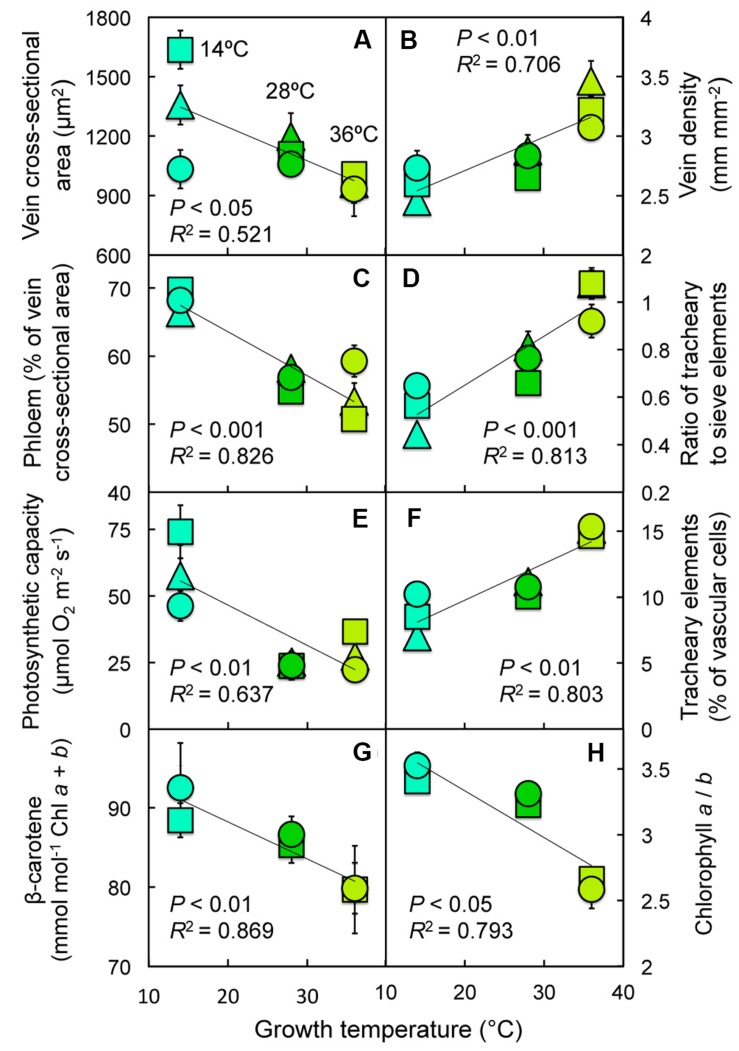
**Relationship between **(A)** cross-sectional area per minor vein, **(B)** minor vein density, **(C)** the fraction of minor vein area comprised of phloem, **(D)** the minor vein ratio of tracheary to sieve elements, **(E)** photosynthetic capacity, **(F)** the fraction of minor veins comprised of tracheary elements, **(G)** the level of β-carotene relative to chlorophyll, or **(H)** the ratio of chlorophyll *a* to *b* and growth temperature during leaf development for leaves of Italian (circles), Col-0 (triangles), and Swedish (squares) *A. thaliana* ecotypes grown at cool (14°C = blue-green symbols), moderate (28°C = green symbols), or hot (36°C = olive-green symbols) leaf temperature**.

**FIGURE 8:**
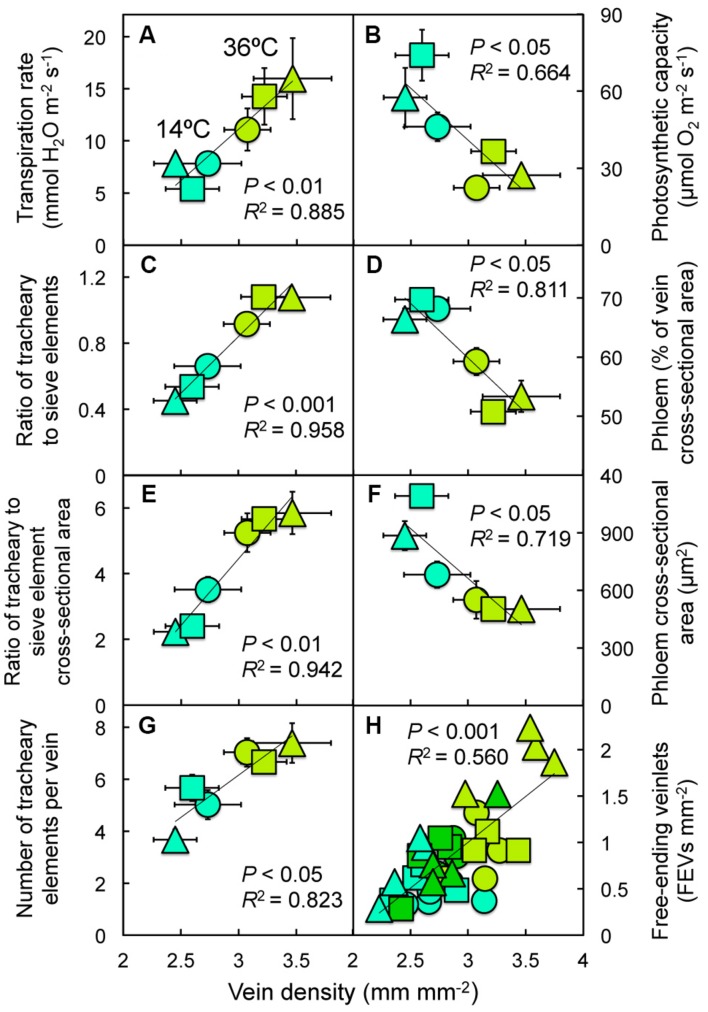
**(A) Transpiration rate, **(B)** photosynthetic capacity, **(C)** the minor vein ratio of tracheary to sieve elements, **(D)** the fraction of minor vein area comprised of phloem, **(E)** the minor vein ratio of tracheary to sieve element cross-sectional area, **(F)** the cross-sectional area of minor veins comprised of phloem, **(G)** the number of tracheary elements per minor vein, and **(H)** the number of free-ending veinlets per unit leaf area for leaves of Italian (circles), Col-0 (triangles), and Swedish (squares) *A. thaliana* ecotypes grown at cool (14°C = blue-green symbols) or hot (36°C = olive-green symbols) and, for **(H)**, moderate (28°C = green) leaf temperature as a function of minor vein density**.

**FIGURE 9:**
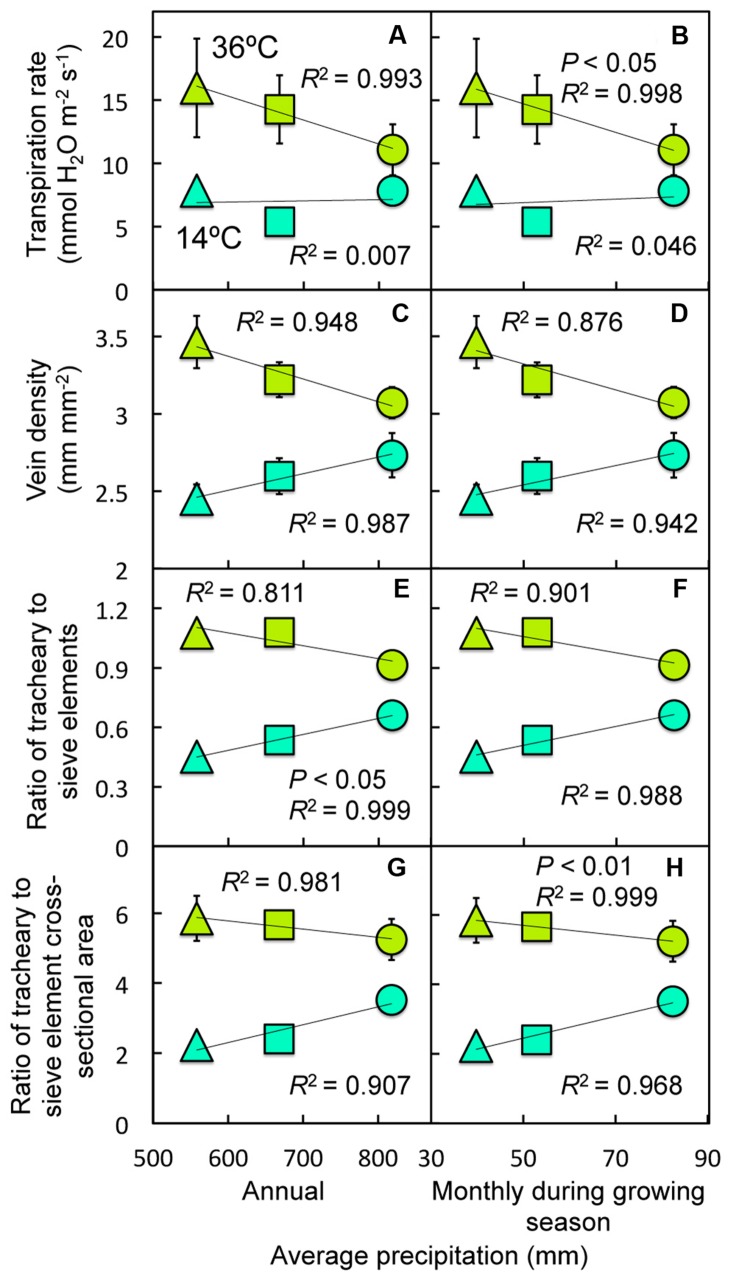
**(A,B) Transpiration rate, **(C,D)** minor vein density, **(E,F)** the ratio of minor vein tracheary to sieve elements, and **(G,H)** the ratio of minor vein tracheary to sieve element cross-sectional area for leaves of Italian (circles), Col-0 (triangles), and Swedish (squares) *A. thaliana* ecotypes grown at cool (14°C = blue-green symbols) or hot (36°C = olive-green symbols) leaf temperature as a function of the average **(A,C,E,G)** annual precipitation and **(B,D,F,H)** monthly precipitation during the growing season at the site from which each ecotype was obtained**.

**FIGURE 10:**
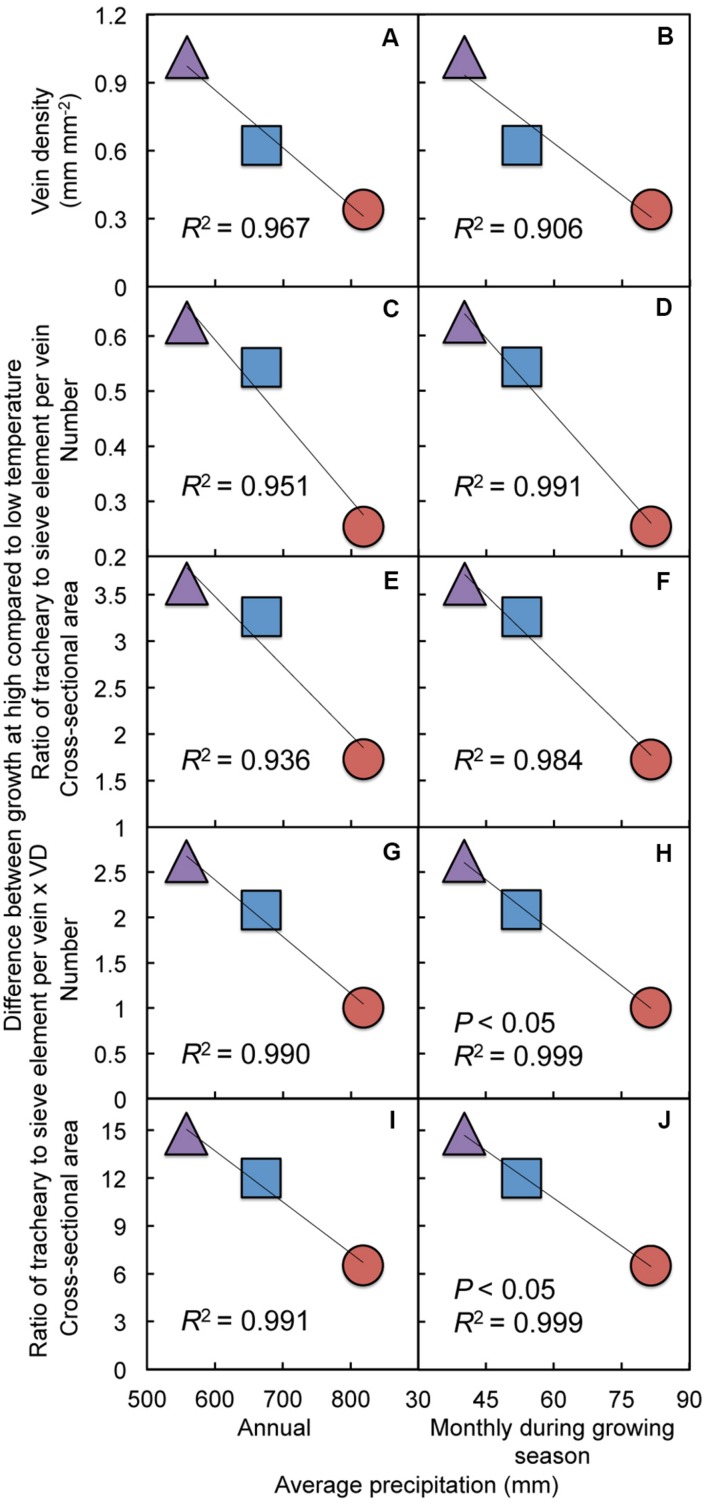
**Differences between growth at high (36°C) compared to low (14°C) temperature in **(A,B)** minor vein density, **(C,D)** the minor vein ratio of tracheary to sieve elements, **(E,F)** the minor vein ratio of tracheary to sieve element cross-sectional area, **(G,H)** the product of the difference in vein density (VD) and the difference in the ratio of tracheary to sieve elements in minor veins, and **(I,J)** the product of the difference in vein density (VD) and the difference in the ratio of tracheary to sieve element cross-sectional area in minor veins for leaves of Italian (red circles), Col-0 (purple triangles), and Swedish (blue squares) *A. thaliana* ecotypes as a function of the average **(A,C,E,G,I)** annual precipitation and **(B,D,F,H,J)** monthly precipitation during the growing season at the site from which each ecotype was obtained**.

**FIGURE 11:**
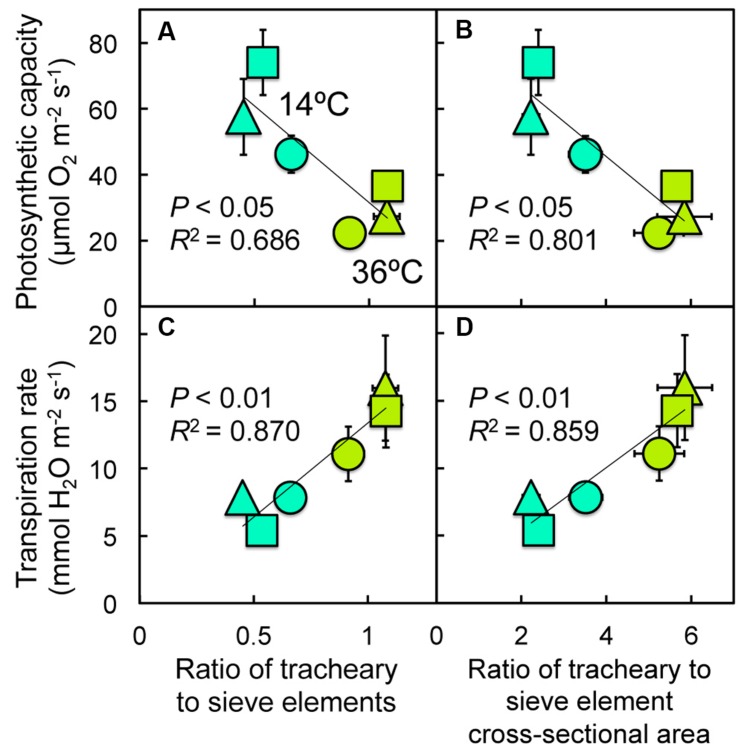
**Relationship between **(A,B)** photosynthetic capacity or **(C,D)** transpiration rate and the ratio of **(A,C)** tracheary to sieve elements per minor vein or **(B,D)** tracheary to sieve element cross-sectional area per minor vein for leaves of Italian (circles), Col-0 (triangles), and Swedish (squares) *A. thaliana* ecotypes grown at cool (14°C = blue-green symbols) or hot (36°C = olive-green symbols) leaf temperature**.

**FIGURE 12:**
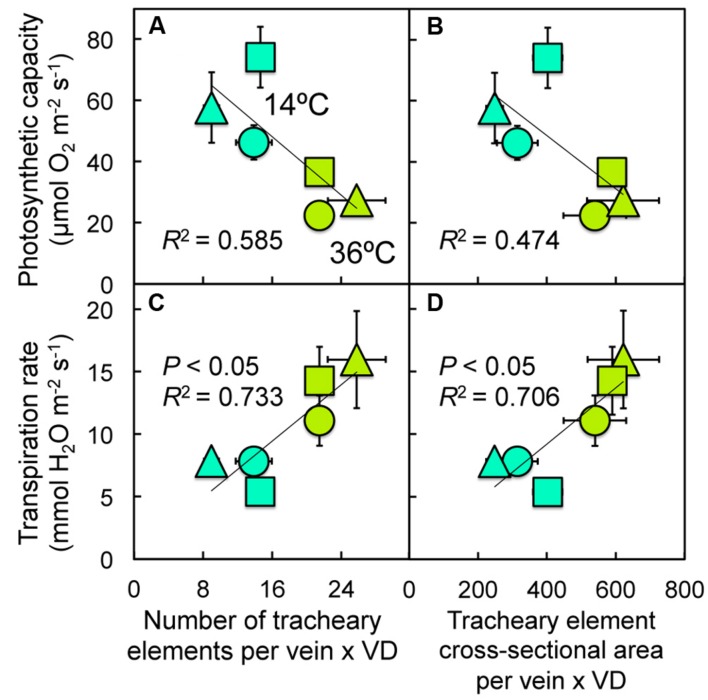
**Relationship between **(A,B)** photosynthetic capacity or **(C,D)** transpiration rate and the product of minor vein density (VD) and **(A,C)** the number of tracheary elements per foliar minor vein or **(B,D)** the cross-sectional area of the minor veins comprised of tracheary elements for leaves of Italian (circles), Col-0 (triangles), and Swedish (squares) *A. thaliana* ecotypes grown at cool (14°C = blue-green symbols) or hot (36°C = olive-green symbols) leaf temperature**.

Vein density (mm vein length per mm^2^ leaf area), vein architectural features (phloem parenchyma [transfer] cell wall ingrowths and numbers and cross-sectional areas of the different xylem and phloem cells), leaf thickness, pigment levels (high pressure liquid chromatography; Shimadzu Corporation, Kyoto, Japan), photosynthetic capacity (light- and CO_2_-saturated rate of photosynthetic oxygen evolution at 25°C; Hansatech oxygen electrode systems, King’s Lynn, Norfolk, UK), and transpiration rate (LCi Portable Photosynthesis System, ADC Bioscientific Ltd., Hoddesdon, Herts, England, UK) during exposure to the growth photon flux density of 400 μmol m^-2^ s^-1^ at a leaf temperature of 28.7 ± 1.6°C (mean ± SD; *n* = 29) and a vapor pressure deficit of 2.74 ± 0.07 kPa (mean ± SD; *n* = 29) were determined as described in Amiard et al. (2005), Dumlao et al. (2012), Cohu et al. (2013a,b), Adams et al. (2014a), and Stewart et al. (2015, 2016). Vein metric analysis was limited to the minor veins (3rd and 4th order veins) as described in detail in Cohu et al. (2013a,b). Photosynthetic oxygen evolution capacity in a water-saturated atmosphere was utilized instead of CO_2_ exchange because the former is unaffected by multiple resistances to CO_2_ diffusion (e.g., stomatal, cuticular, mesophyll, chloroplastic) into the sites of carboxylation (Delieu and Walker, 1981), thus providing a measure of maximal electron transport capacity to match the various vascular metrics that serve as proxies for the capacity to load and export sugars from the leaves. The moderate conditions chosen for determination of transpiration rate proved to be sufficient to elucidate the variation present among the ecotypes that developed under the different growth temperature regimes without inducing stomatal closure that might result from employment of a higher light intensity, higher temperature, or greater vapor pressure difference between the leaves and the atmosphere. Some of the data presented are derived from the experiments described in Cohu et al. (2013a,b), Adams et al. (2014a), and Stewart et al. (2016), although many of the metrics have not been evaluated previously (e.g., leaf thickness, pigment data) or comprehensively across all three ecotypes in the context of latitude and habitat environmental conditions, and data from Col-0 grown under high temperature were not included in any of the previous studies. Vein densities (*n* = 3 to 4 plants), photosynthetic capacities (*n* = 3 to 4 plants), transpiration rates (*n* = 4 to 5 plants), and pigment data (*n* = 3 to 4 plants) are presented as mean values ± SD (with the exception that **Figure 8H** represents a scatter plot of all data points), whereas leaf thickness (three cross-sections from each of 3 to 4 plants) and all of the vein architectural features (*n* = 7 to 10 minor veins per plant from 3 to 4 plants) are presented as mean values ± SE. With the exception of **Figures 5** and **10** that depict the calculated difference between metrics from plants grown under the low compared to the high temperature regimes and utilize the country of origin color coding (see **Figure 1**), the symbols in **Figures 4–12** are color coded according to the foliar chlorophyll *a*/*b* ratio and resulting subtle differences in visual appearance of the plants grown under the different temperature regimes (**Figure 7H**): blue-green (highest chlorophyll *a/b* ratio) for the low growth temperature regime, green (intermediate chlorophyll *a/b* ratio) for the intermediate growth temperature regime, and olive-green (lowest chlorophyll *a/b* ratio) for the high growth temperature regime.

### Statistical Analyses

Data were subjected to linear regression analysis and two-way analysis of variance for comparison of multiple means to discriminate between the effects of growth temperature, ecotype, and response of the ecotypes to temperature using Pro 11.0.1 JMP software (SAS Institute Inc., Cary, NC, USA).

## Results

In their respective original habitats, the Italian ecotype experiences the smallest annual variation in photoperiod (from just over 9 h of daylight on the winter solstice to about 14 h at the end of seed set in April) during an annual life cycle, whereas the Swedish ecotype experiences photoperiods ranging from just under 5 h in December to 20 h at the end of June (**Figures 2A–C**). We estimate that the Col-0 ecotype germinates from mid-September through October, overwinters as a rosette, and flowers and sets seed in April and May, and thus experiences photoperiods intermediate to those of the Italian and Swedish ecotypes ranging from about 7.6 h at winter solstice to close to 17 h at the end of its life cycle. The range of temperatures experienced by each ecotype during the growing season in its native habitat increases with increasing latitude, from a differential between mean monthly minima and maxima of 18°C (2.7°C to 20.6°C) at the Italian site, to 24.7°C (-5.0°C to 19.7°C over the estimated growing period) at the Polish site, and 31°C (-12.6°C to 18.4°C) at the Swedish site (**Figures 2D–F**). Average annual temperature maxima and minima in the three sites vary linearly with latitude (**Figure 3A**). Precipitation patterns, on the other hand, do not follow a latitudinal gradient (**Figures 2G–I** and **3B**), but nonetheless present a range of variation among the habitats of origin: during the respective growing seasons, the habitat of the Italian ecotype receives the most precipitation (577 mm over seven months), followed by the Swedish site (529 mm over 10 months), with the Polish site receiving the least precipitation (362 mm over the estimated growing period of nine months).

Photosynthetic capacity (**Figures 4A,B**) and leaf thickness (**Figures 4C,D**) of the three ecotypes grown in climate-controlled growth chambers under two different experimental temperature regimes increased linearly with either increasing latitude of origin (**Figures 4A,C**) or decreasing average annual temperature in each respective habitat of origin (**Figures 4B,D**), particularly for cool-grown leaves. These linear increases in photosynthetic capacity and leaf thickness for the cool-grown leaves were paralleled by linear increases in several features of the foliar phloem, including the cross-sectional area per minor vein of the sugar-loading and -exporting sieve elements (**Figures 4E,F**), the number of phloem cells per minor vein that facilitate sucrose loading into sieve elements (**Figures 4G,H**), and increases in cell membrane length (and presumably membrane area) resulting from invagination of phloem parenchyma cell walls (**Figures 4I,J**). In contrast to the responses of cool-grown leaves, hot-grown leaves did not display such increases in phloem features (**Figures 4E–J**). There were also significant ecotypic differences in photosynthetic capacity, leaf thickness, and sieve element cross-sectional area per vein, significant ecotype × temperature interactions for the latter two parameters as well as for the number of loading cells per minor vein, and all of these features varied significantly with growth temperature in controlled growth chambers (**Table 1**).

**Table 1:** Results of two-way analysis of variance (ANOVA) for the effect of ecotype, growth temperature, and the degree of ecotype response to growth temperature (14°C and 36°C) for all data presented, with statistically significant effects indicated by asterisks (^∗^*P* < 0.05; ^∗∗^*P* < 0.01; ^∗∗∗^*P* < 0.001; *n.s.*, not significant).

Metric	Ecotype	Temperature	Ecotype × temperature
Photosynthetic capacity (μmol O_2_ m^-2^ s^-1^)	^∗∗∗^	^∗∗∗^	*n.s.*
Leaf thickness (μm)	^∗∗∗^	^∗∗∗^	^∗^
Sieve element cross-sectional area per minor vein (μm^2^)	^∗∗∗^	^∗∗∗^	^∗∗∗^
Loading cell number per minor vein	*n.s.*	^∗∗∗^	^∗∗^
% increase in plasma membrane due to wall ingrowth	*n.s.*	^∗∗∗^	*n.s.*
Minor vein cross-sectional area (μm^2^)	^∗^	^∗∗∗^	*n.s.*
Minor vein phloem cross-sectional area (μm^2^)	*n.s.*	^∗∗∗^	^∗^
Minor vein xylem cross-sectional area (μm^2^)	^∗^	*n.s.*	*n.s.*
Foliar minor vein density (mm mm^-2^)	*n.s.*	^∗∗∗^	^∗^
Phloem (% of minor vein cross-sectional area)	*n.s.*	^∗∗∗^	*n.s.*
Ratio of tracheary to sieve elements in minor veins	*n.s.*	^∗∗∗^	^∗∗^
Tracheary elements (% of minor vein vascular cells)	^∗^	^∗∗∗^	*n.s.*
β-carotene (mmol mol^-1^ Chl *a* + *b*)	*n.s.*	^∗∗∗^	*n.s.*
Chlorophyll *a*/*b*	*n.s.*	^∗∗∗^	*n.s.*
Transpiration rate (mmol H_2_O m^-2^ s^-1^)	*n.s.*	^∗∗∗^	^∗^
Ratio of tracheary to sieve element cross-sectional area	*n.s.*	^∗∗∗^	*n.s.*
Number of tracheary elements per minor vein	*n.s.*	^∗∗∗^	*n.s.*
Free-ending veinlets (FEVs mm^-2^)	^∗∗∗^	^∗∗∗^	^∗^
Number of tracheary elements per minor vein × VD	*n.s.*	^∗∗∗^	^∗^
Tracheary element cross-sectional area per vein × VD	*n.s.*	^∗∗∗^	*n.s.*


*VD = vein density.*

When expressed as the difference between hot-grown and cool-grown leaves (as a measure of the degree of responsiveness to experimental growth temperature under controlled conditions), photosynthetic capacity, leaf thickness, and minor vein phloem metrics exhibited linear increases with increasing latitude and also with decreasing temperature in the habitat of origin (**Figure 5**). While linear regressions for photosynthetic capacity (**Figures 5A,B**) and cross-sectional area of sieve elements comprising the minor veins (**Figures 5E,F**) with latitude or average annual temperature at ecotype origin were significant, the phloem features of phloem cell numbers and cell wall ingrowth levels, together serving as proxies for total phloem cell membrane area, were not. In particular, leaves of Col-0 exhibited levels of phloem transfer cell wall ingrowths that fell slightly below the line (**Figures 5I,J**) and numbers of phloem loading cells (**Figures 5G,H**) that fell slightly above the line; it is thus attractive to speculate that these two features together do effect a level of total phloem cell membrane area adjustment in the Col-0 ecotype in line with that of the other two ecotypes.

Foliar minor vein cross-sectional area in (experimentally) cool-grown leaves increased linearly with increasing latitude (**Figure 6A**) and decreasing annual temperature in the respective habitats of origin (**Figure 6B**), and this was due to increases in the cross-sectional areas of both the phloem (**Figures 6C,D**) and xylem (**Figures 6E,F**). Both of the latter metrics varied significantly as a result of growth temperature, while minor vein cross-sectional area also varied significantly among ecotypes and the phloem cross-sectional area showed significant ecotype × temperature interactions (**Table 1**). However, for (experimentally) hot-grown leaves, only the cross-sectional area of the xylem increased with increasing latitude (**Figure 6E**) and decreasing annual temperature (**Figure 6F**) in the respective habitats of origin; these latter trends did not differ between (experimentally) cool- and hot-grown leaves, but there was a significant difference in xylem cross-sectional area among the ecotypes (**Table 1**).

Furthermore, a number of foliar features also exhibited significant, linear relationships with temperature during (experimental) plant growth across ecotypes (**Figure 7**; **Table 1**), with some features exhibiting positive and others negative correlations. While cross-sectional area of the minor veins decreased with increasing growth temperature under controlled experimental conditions (**Figure 7A**), due in large part to decreases in phloem tissue (**Figure 7C**), vein density (minor vein length per leaf area) increased with increasing growth temperature (**Figure 7B**), as did the ratio of tracheary to sieve elements in minor veins (**Figure 7D**) and the fraction of vascular cells per minor vein represented by tracheary elements (**Figure 7F**). Three additional parameters decreased with increasing growth temperature: photosynthetic capacity (**Figure 7E**), β-carotene levels (**Figure 7G**), and chlorophyll *a*/*b* ratio (**Figure 7H**). In addition to (experimental) growth temperature having a highly significant impact on all of these features, the fraction of vascular cells comprised of tracheary elements was significantly different among ecotypes, and vein density and the minor vein ratio of tracheary to sieve elements exhibited significant ecotype × temperature interactions (**Table 1**).

When evaluated under a common set of experimental conditions, transpiration rate was significantly influenced by the temperature of the plants during growth, and also displayed significant ecotype × temperature interactions (**Table 1**). When combining data for leaves of the three ecotypes grown under either low or high temperature in controlled conditions, transpiration rate was found to increase with increasing vein density (**Figure 8A**), as did several minor vein xylem features, including ratios of either the numbers (**Figure 8C**) or the cross-sectional areas of tracheary to sieve elements (**Figure 8E**), and the number of tracheary elements per minor vein (**Figure 8G**), all of which were significantly influenced by (experimental) growth temperature (**Table 1**). On the other hand, photosynthetic capacity (**Figure 8B**), the fraction of minor veins occupied by phloem (**Figure 8D**), and the cross-sectional area of minor vein phloem (**Figure 8F**) decreased linearly with increasing vein density. One vascular feature that may contribute to the variation in foliar vein density is the level of free-ending veinlets, of which the greatest range of phenotypic plasticity (greatest difference between cool- and hot-grown leaves) was seen in the Col-0 ecotype (**Figure 8H**) and that also varied significantly among ecotypes, with (experimental) growth temperature, and with ecotype × temperature interactions (**Table 1**). One intriguing feature of these relationships is that, for vein density as well as the ratios of tracheary to sieve element number and area (**Figures 8C,E**), the Italian ecotype (circles) exhibited the smallest difference between (experimentally) cool- and hot-grown leaves, the Swedish ecotype (squares) exhibited an intermediate difference, and the Col-0 ecotype (triangles) exhibited the greatest difference.

In contrast to photosynthetic capacity and foliar minor vein phloem features of plants grown under controlled conditions, which varied with latitudinal and temperature gradients between the habitats of origin (**Figures 4**–**6C,D**), transpiration rate, foliar minor vein density, and the minor vein ratio of tracheary to sieve element numbers and cross-sectional areas of plants grown under controlled conditions did not vary regularly with latitude or average temperatures for the respective sites from which each ecotype originated (relationships not shown). Therefore, these latter parameters were instead evaluated for a possible association with variation in precipitation levels among the three sites of origin. For (experimentally) hot-grown leaves of the three ecotypes, transpiration rate (**Figures 9A,B**), vein density (**Figures 9C,D**), and tracheary to sieve element ratios (**Figures 9E–H**) exhibited trends for decreases with increasing levels of habitat-of-origin precipitation, whereas for (experimentally) cool-grown leaves all three vascular features exhibited trends for increases with increasing levels of habitat-of-origin precipitation level (**Figures 9C–H**). Moreover, the extent of phenotypic plasticity (difference between cool- and hot-grown leaves) of the latter three vascular metrics was *negatively* correlated with average precipitation (annual and monthly during the growing season) in the respective habitats of origin (**Figures 10A–F**). Intriguingly, when normalized for differences in vein density – by multiplying the difference in vascular cell numbers or cross-sectional area by the difference in vein density (VD) – the correlations between average precipitation in the respective habitats of origin and the extent of the differences in tracheary to sieve element ratios between (experimentally) cool- and hot-grown leaves became stronger yet (**Figures 10G–J**) and significant for average monthly precipitation during the growing season in the habitat of origin (**Figures 10H,J**).

While the capacity for photosynthetic oxygen evolution exhibited *negative* linear relationships with the minor veins’ ratio of tracheary to sieve element numbers (**Figure 11A**) and cross-sectional areas (**Figure 11B**) as well as with the product of vein density and (**Figure 12A**) tracheary element number or (**Figure 12B**) tracheary element cross-sectional area of minor veins, transpiration rate was significantly and *positively* correlated with all of these tracheary element features (**Figures 11C,D** and **12C,D**).

## Discussion

For an herbaceous annual that overwinters as a rosette and persists through the summer as a seed, an elevated photosynthetic capacity in cool-grown versus warm-grown leaves presumably allows such a species to take full advantage of cool autumn, winter, and spring days for photosynthesis. Consistent with previous studies of *A. thaliana* (Adams et al., 2013a, 2014a; Cohu et al., 2013b, 2014b; Stewart et al., 2016), photosynthetic capacity of plants grown under controlled conditions was closely correlated with a variety of foliar phloem metrics that relate to the loading of sucrose into the phloem and its export from the leaf. A greater cross-sectional area of sieve elements in minor veins of (experimentally) cool-grown leaves of Col-0, and especially of the Swedish ecotype, may aid in the flux of phloem sap that becomes more viscous at the lower temperatures experienced by these ecotypes in their natural habitats (see discussion in Cohu et al., 2013a). On the other hand, the Italian ecotype exhibited no adjustment in minor vein sieve element cross-sectional area in cool-grown leaves, which would be consistent with the assumption that low-temperature-induced increases in viscosity rarely constitute an impediment to foliar sugar export in the Italian ecotype in its warmer natural habitat. Features of the minor vein phloem that increase the cell membrane area available for placement of transport proteins (sucrose efflux proteins in phloem parenchyma cells, ATPases in phloem parenchyma and companion cells, and proton-sucrose co-transporters in companion cells and sieve elements; see Haritatos et al., 2000; Adams et al., 2014a; Duan et al., 2014), including cell number and the level of cell wall ingrowths in phloem parenchyma cells, exhibited a latitudinal trend in cool-grown leaves that paralleled that of photosynthesis across the three ecotypes. Since, among the phloem metrics analyzed, it was only the level of cell wall ingrowth that differed between cool- and hot-grown leaves of the Italian ecotype, one may speculate that cell wall modification may be sufficient to provide the additional level of sucrose feeding and proton pumping into the apoplast to support the modest level of additional photosynthetic activity seen in cool-grown leaves of the Italian versus the Swedish ecotype. For additional discussion of possible cell wall ingrowth functions, see Amiard et al. (2007) and Adams et al. (2014a).

The concomitant upregulation of photosynthetic capacity and greater leaf thickness in response to experimental growth at low compared to elevated temperature under controlled conditions, as well as the trends for increasing photosynthetic capacity paralleled by increasing leaf thickness with increasing latitude and decreasing temperature for the habitat from which each ecotype originated, are consistent with similar low-temperature induced acclimatory adjustments in leaf thickness and photosynthetic capacity in the winter annual spinach (Dumlao et al., 2012) and the biennial *Verbascum phoeniceum* (Dumlao et al., 2012; Muller et al., 2014b). Increasing the volume of mesophyll tissue per unit leaf area presumably facilitates an increase in the number of chloroplasts that contribute to photosynthesis. The fact that, in addition to multiple phloem metrics, greater levels of foliar β-carotene and higher chlorophyll *a*/*b* ratios paralleled the elevated photosynthetic capacity in cool-grown leaves is consistent with a greater emphasis on reaction centers and electron transport components in cool-grown leaves. Leaves with higher photosynthetic capacities would be expected to feature a greater ratio of reaction centers, where β-carotene and chlorophyll *a* are located, relative to the outer light-harvesting proteins, where chlorophyll *b* is localized (Anderson and Osmond, 1987; Yamamoto and Bassi, 1996).

One can speculate that the trends for vein density to increase with increasing growth temperature (due at least in part to proliferation of free-ending veinlets), and for the number of tracheary elements per vein to increase with increasing vein density, may contribute to an increased capacity to deliver water to the leaves in support of higher levels of transpirational cooling in hot-grown leaves. The absence of a positive association between these xylem features and photosynthesis contrasts with associations observed between foliar hydraulic conductance, vein density, and photosynthesis in studies of other species (Hubbard et al., 2001; Santiago et al., 2004; Brodribb et al., 2005, 2007, 2010; Nardini et al., 2005; Sack and Holbrook, 2006; Maherali et al., 2008; Boyce et al., 2009; Beerling and Franks, 2010; Brodribb and Feild, 2010; McKown et al., 2010; Blonder et al., 2011; Walls, 2011; Zhu et al., 2013). While we previously obtained positive correlations between xylem features and photosynthesis for several summer annual crop species grown under a single controlled growth temperature (Muller et al., 2014a), we obtained only weak, mostly non-significant associations for *A. thaliana* grown under two growth temperatures and two photon flux densities (and only by excluding the data from the Col-0 ecotype; Cohu et al., 2013b). It is possible that such differences may result from differences in the demand for water transport versus photosynthesis in summer versus winter (and in summer versus winter annuals). For a winter annual, such as *A. thaliana*, that is active during the colder months of the year and responds to low temperature with upregulation of photosynthesis, the system for transport and distribution of water to and within the leaves may not be expected to parallel photosynthesis if evaporative demand is relatively low at the cool temperatures. On the other hand, leaves of summer annuals and winter-deciduous perennial species may experience higher temperatures and greater evaporative demand during their seasonal peak periods of photosynthetic activity, and concordance among foliar phloem and xylem metrics, hydraulic conductance, transpiration, and photosynthesis may be common (Muller et al., 2014a).

The different response of vein density and photosynthetic capacity to leaf growth environment in *A. thaliana* may also be associated with possible differences in response between summer and winter annuals. While foliar vein density and photosynthetic capacity exhibited concomitant upregulation in a summer annual and two biennial species (Amiard et al., 2005; Muller et al., 2014b), winter annuals (including *A. thaliana*) exhibited upregulation of photosynthesis with no difference in vein density in certain controlled environments (Amiard et al., 2005; Cohu et al., 2013b) or an upregulation of vein density in hot-grown leaves associated with a downregulation of photosynthesis (Stewart et al., 2016). Moreover, as shown here, the extent of the adjustment in vein density in response to experimental growth temperature under controlled conditions varied strongly among *A. thaliana* ecotypes: vein density adjustments were least pronounced in the Italian ecotype, followed by the Swedish ecotype (vein densities in cool-and hot-grown leaves bracketing those of the Italian ecotype), with vein density exhibiting the greatest differential response in the Col-0 ecotype (bracketing that of the Swedish ecotype), a pattern that did not follow the latitudinal and temperature gradients of the ecotypes’ respective habitats.

Rather than with temperatures at the respective habitats of origin, the extent of vein density adjustments to experimental growth temperature was inversely correlated with average precipitation in the habitat from which each ecotype originated, as was the extent of the adjustment in the ratio of tracheary to sieve elements to experimental growth temperature (particularly when the latter metrics were normalized for adjustments in vein density). Moreover, the number of tracheary elements per minor vein and transpiration rate were both positively correlated with vein density. These findings suggest that (i) water delivery to the leaves is enhanced not only by a greater foliar vein density but also by a greater number of tracheary elements per vein and (ii) the ability to adjust vein density and the number of tracheary elements per vein to growth at hot temperature is particularly important in natural habitats with lower precipitation levels. Under low temperature conditions, the gradient in water potential from leaf to the atmosphere is relatively low, and the needs of the phloem could presumably be met by a lower tracheary to sieve element ratio, whereas a greater gradient in water potential from the leaf to the atmosphere at high growth temperature presumably necessitates a higher tracheary to sieve element ratio. While water delivery to mesophyll and epidermal cells of the leaf is one important function of xylem conduits, another function of tracheary elements is delivery of water to the phloem to facilitate the pressure-driven flow of viscous, sugar-laden sap in sieve elements from leaves to the plant’s sinks (Boersma et al., 1991; Patrick et al., 2001; Hölttä et al., 2006; Sevanto et al., 2011; Hölttä and Nikinmaa, 2013; Nikinmaa et al., 2013). For adequate phloem pressurization to drive sugar transport from source leaves to sinks, the water potential gradient from xylem to phloem must be greater than that from xylem to the air when stomates are open (Nikinmaa et al., 2013), and this gradient between xylem and phloem is the primary driver of water flow in the xylem when stomata are closed.

A growing body of evidence supporting a relationship between foliar phloem features that facilitate the export of reduced carbon compounds from the leaf and photosynthesis has been generated over the past decade (Adams et al., 2005, 2007, 2013a, 2014a; Amiard et al., 2005; Ainsworth and Bush, 2011; Cohu et al., 2013a,b, 2014b; Hölttä and Nikinmaa, 2013; Nikinmaa et al., 2013; Muller et al., 2014a,b; Stewart et al., 2016). Given that mesophytic annual and biennial species exhibit an upregulation of photosynthesis in response to growth at low compared to moderate or high temperature (Holaday et al., 1992; Adams et al., 1995, 2001a,b, 2002, 2004, 2013a; Martindale and Leegood, 1997; Strand et al., 1997; Verhoeven et al., 1999; Dumlao et al., 2012), it is perhaps not surprising that the extent of photosynthetic upregulation, as well as the associated capacity of the phloem for sugar transport, follows the temperature cline among the three *A. thaliana* ecotypes in the present study. Such upregulation presumably counteracts the impact of low temperature on the activity of Calvin cycle enzymes, the activity of the membrane-spanning transport proteins, and the viscosity of the phloem sap (Cohu et al., 2013a,b, 2014b), permitting each ecotype to fix CO_2_ and export sugars from the leaves despite low temperatures as long as liquid water is available. The finding that the Swedish ecotype, that experiences the lowest temperatures in its native growing season, upregulates photosynthesis and foliar phloem features to the greatest extent in response to (experimental) growth under low temperature, while the Polish ecotype that experiences intermediate temperatures and the Italian ecotype that experiences the relatively highest temperatures during their respective growing seasons exhibit intermediate and lower levels of such upregulation, respectively, are consistent with this interpretation.

Studies that focused on leaf features have revealed relationships that are consistent with the findings of the current study with regard to water availability. Two major overviews showed that leaf vein density (which is often positively associated with leaf hydraulic conductivity; Sack and Scoffoni, 2013) is strongly negatively correlated with precipitation level and positively associated with increasing aridity (Uhl and Mosbrugger, 1999; Sack and Scoffoni, 2013). Moreover, in a study that is most comparable to the current investigation, growth of six Hawaiian species of the herbaceous genus *Plantago* under common conditions (plus a seventh characterized in the field) revealed that species from drier habitats had higher foliar vein densities (Dunbar-Co et al., 2009), consistent with the findings reported here. On the other hand, hydraulic conductivity and/or xylem features associated with hydraulic conductivity have generally been found to be positively associated with increasing levels of precipitation in the stems and trunks of woody shrubs and trees (Olano et al., 2012; Pratt et al., 2012; Abrantes et al., 2013; Gea-Izquierdo et al., 2013; Castagneri et al., 2015; Schreiber et al., 2015; Venegas-González et al., 2015; Pfautsch et al., 2016; but see Barnard et al., 2011). Furthermore, two previous studies on plants from sites differing in temperature and precipitation found opposite relationships to those observed here for the three ecotypes of *A. thaliana*. Creese et al. (2011) grew 26 *Pinus* species under common greenhouse conditions and Zhu et al. (2012) grew seven populations of the oak *Quercus variabilis* (a winter deciduous tree) in a common garden and found that plant hydraulic conductivity was not correlated with precipitation level but instead with increasing temperature of the habitat from which the species or populations originated (see also Fonti et al., 2013).

Perhaps these seemingly divergent findings can be reconciled by consideration of the habitats in which the different species are found and their diverging strategies for acclimating to the range of conditions to which each species is adapted. Firstly, the habitats to which woody species such as conifers and oaks are adapted may simply extend over a much broader range of aridity (into regions of lower precipitation, lower water availability, and greater evaporative demand) than the habitats in which herbaceous species grow and/or the long-lived nature of woody species may require the latter species to persist through seasonal periods of even lower water availability and greater evaporative demand. Consequently, such woody species may place an emphasis on the prevention of cavitation (through smaller tracheids with lower hydraulic conductivity) with decreasing precipitation level. For herbaceous species, an emphasis may instead be on greater hydraulic conductivity to meet the evapotranspirational demand of the leaves at elevated temperatures. The current findings thus contribute to the debate concerning the trade-off (or lack thereof) between hydraulic efficiency of the xylem versus its susceptibility to cavitation (Maherali et al., 2004; Hacke et al., 2006; Fan et al., 2011; Fichot et al., 2011; Lens et al., 2011; Manzoni et al., 2013; Zhu et al., 2013; Aranda et al., 2015; Kröber et al., 2015; Gleason et al., 2016; Pfautsch et al., 2016). It is also such that woody species tend to utilize mechanisms to ameliorate the heat load on the leaves that also concomitantly reduce water loss (e.g., growth of leaves at angles to reduce light interception at midday, development of thicker, more reflective foliar cuticles; Logan et al., 2014), whereas herbaceous mesophytes often display their leaves to intercept full sunlight at midday to maximize photosynthesis but consequently rely on evaporative cooling to prevent the leaves from overheating. Such a strategy would not, of course, be viable were it not such that the mesophytes occupy sites (in space and/or time) that can provide sufficient water to meet those evaporative requirements. Moreover, the inherent growth rates of long-lived, woody species are typically lower than those of rapidly growing, herbaceous mesophytes (Adams et al., 2014b). Mesophytes thus employ higher rates of photosynthesis (and the concomitant necessity for greater hydraulic conductance to facilitate higher rates of CO_2_ fixation through greater stomatal numbers and/or stomata that are open wider and of carbon export from the leaves that requires more water for the greater turgor-pressure driven movement of sugars through the phloem) to meet their greater sink activity compared to woody species with lower sink activity and rates of photosynthesis.

To briefly summarize, photosynthetic capacity and foliar phloem features presumably indicative of foliar sugar-loading and sugar-export capacity varied in a consistent manner with latitude and temperature of the habitat of origin (greatest in the Swedish ecotype from high latitude and lowest prevailing temperature), whereas vein density and water-transporting tracheary elements varied with precipitation level of the habitat of origin (greatest in the Col-0 ecotype from Poland with the lowest level of precipitation). The nature of these relationships depended on conditions experienced by the plants during growth. The phloem and photosynthesis relationships could be observed in the plants grown under low temperature, whereas the xylem and transpiration relationships could be observed in the plants grown under hot temperature. Moreover, the level of responsiveness in the characterized features was most strongly clarified by comparing the range over which the features acclimated to the two extreme experimental growth temperatures utilized in the present study. Growing all three ecotypes under common conditions at an intermediate temperature revealed very little of the ecotypic differentiation that this species harbors. Similarly, growth of the plants at a higher light intensity masked the extent to which the ecotypes respond to temperature (see Cohu et al., 2013b).

Future studies exploring ecotypic differences within a species should thus utilize more than one common experimental growth condition. The straightforwardness of the relationships illuminated in the current study may have been a result of the consistent nature of the three habitats from which the ecotypes were obtained (all being low altitude sites under the influence of maritime conditions along a north–south transect). Were additional ecotypes from more diverse climatic conditions such as higher altitudes, from inland sites further from major bodies of water, or from sites further to the east in latitude (e.g., from Russia, India, China) or west in latitude (e.g., Spain, UK) to be characterized in the same manner, it is possible that their responses may not align closely with those observed in the present study. Moreover, to further understand the relationships evaluated in the current study, additional species from a range of environments should also be characterized. For instance, it is possible that some species responding to declining winter temperatures through shedding of leaves (winter deciduous) or downregulation of photosynthesis (characteristic of many sclerophyllous evergreen species) may exhibit different relationships between xylem features associated with hydraulic conductivity and habitat temperatures and precipitation levels than herbaceous annual and biennial species that respond to low winter temperatures through upregulation of photosynthesis (Adams et al., 2001a, 2002, 2004, 2006, 2013a,b, 2014a,b). Relationships among vein density, foliar phloem and xylem features, hydraulic conductivity, photosynthesis, and transpiration should thus be characterized in the context of growth form (e.g., herbaceous versus woody, mesophytic versus sclerophytic), life span and habit (e.g., annual versus biennial versus perennial, deciduous versus evergreen), major taxonomic groups (e.g., ferns versus gymnosperms versus angiosperms, monocots versus dicots), mode of phloem loading (apoplastic versus symplastic versus non-loading species), and habitat, e.g., across sites that vary in precipitation but not temperature, across sites that vary in temperature but not precipitation, and across sites where temperature and precipitation vary in the same direction or the opposite direction.

## Author Contributions

BD-A and WA designed the experiments, CC, OM, and JS carried out the experiments and collected the data, JS assembled the climatological data, executed the statistical analyses, and rendered the figures, and WA and BD-A analyzed the data and wrote the manuscript, with input from JS.

## Conflict of Interest Statement

The authors declare that the research was conducted in the absence of any commercial or financial relationships that could be construed as a potential conflict of interest.

## Footnotes

1 https://www.arabidopsis.org

2 http://aa.usno.navy.mil/data/docs/Dur_OneYear.php
